# Clinical mobility metrics estimate and characterize physical activity following lower-limb amputation

**DOI:** 10.1186/s13102-022-00518-x

**Published:** 2022-07-07

**Authors:** Mayank Seth, Ryan Todd Pohlig, Gregory Evan Hicks, Jaclyn Megan Sions

**Affiliations:** 1 Delaware Limb Loss Studies, Department of Physical Therapy, University of Delaware, 540 South College Avenue, Suite 210JJ, Newark, DE 19713 USA; 2 Biostatistics Core, University of Delaware, Newark, DE USA; 3 Delaware Spine Studies, Department of Physical Therapy, University of Delaware, Newark, DE USA

**Keywords:** Prosthesis, Physical inactivity, Outcome measures, Walking speed, Rehabilitation, Walk test, Fitness trackers

## Abstract

**Background:**

Regular physical activity following a lower-limb amputation is essential for maintaining health and a high quality of life. Most adults with a lower-limb amputation, however, participate in insufficient daily physical activity, and thus, are predisposed to poor health outcomes. Estimating physical activity after lower-limb amputation via common mobility metrics may aid in clinical decisions regarding treatment prioritization and prosthesis prescription. The objectives of this study were (a) to examine associations between daily physical activity and patient-reported and performance-based mobility metrics among adults with lower-limb amputation, and (b) to determine whether patient-reported and performance-based mobility metrics can distinguish between physical activity status [i.e., sedentary (< 5000 steps/day) or non-sedentary (≥ 5000 steps/day)] of adults with lower-limb amputation.

**Methods:**

A cross-sectional study involving 35 adults with a unilateral transtibial (N = 23; 63.0 ± 10.4 years) or transfemoral amputation (N = 12; 58.8 ± 9.5 years) was conducted. Participants completed patient-reported (Prosthesis Evaluation Questionnaire-Mobility Subscale) and performance-based mobility metrics (L-Test, 10-m Walk Test, 6-min Walk Test). Physical activity, i.e., average steps/day, was measured with an accelerometer.

**Results:**

Patient-reported and performance-based mobility metrics were associated with daily physical activity (*p* < 0.050). Prosthesis Evaluation Questionnaire-Mobility Subscale scores, L-Test time, 10-m Walk Test speed and 6-min Walk Test distance independently explained 11.3%, 31.8%, 37.6% and 30.7% of the total variance in physical activity. Receiver operating characteristic curves revealed patient-reported and performance-based mobility metrics significantly distinguish between physical activity status, i.e., sedentary (< 5000 steps/day) versus non-sedentary (≥ 5000 steps/day). Preliminary cut-points for mobility metrics to classify physical activity status were determined.

**Conclusions:**

Following a lower-limb amputation, patient-reported and performance-based mobility metrics may estimate daily physical activity, thereby aiding clinical decisions regarding treatment prioritization as well as prosthesis selection.

## Background

Following a lower-limb amputation (LLA), to promote health and maintain a high quality of life, engaging in regular physical activity is recommended [1, 2]. Current evidence, however, indicates only about 40% of adults with lower-limb amputation (LLA) participate in sufficient daily physical activity [3, 4]. Hence, a large proportion of adults with LLA may be predisposed to adverse health outcomes, including progressive loss in physical function, development or worsening of chronic health conditions (e.g., cardiovascular disease, diabetes mellitus) [5], and increased risk of morbidity and mortality [6]. Given current physical activity trends, alongside an estimated growth in this patient population to 3.6 million by 2050 [7], increasing burden to the healthcare systems is anticipated.

Clinically, the ability to estimate a patient’s daily physical activity (i.e., steps taken during various daily locomotor activities [8]) can aid in treatment prioritization, as well as enhance intervention effectiveness and prosthesis prescription. For example, clinician awareness of a patient’s physical activity level may result in not only altered intervention selections, such as inclusion of aerobic training, but also altered communication, e.g., inclusion of motivational interviewing and/or counseling to overcome physical activity barriers and promote lifestyle changes [9]. Post-amputation, as activity level is an important consideration in prosthesis prescription [10, 11], estimating daily physical activity may allow matching of prosthetic componentry to patient activity requirements.

While physical activity among adults with LLA can be accurately gathered with commercially-available accelerometers that capture daily stride counts [12], financial, as well as data acquisition and processing costs, challenge clinical practice feasibility. Conversely, physical activity estimates obtained via clinical mobility measures may be an inexpensive, quick, objective, and accurate means of estimating physical activity post-LLA and particularly appealing to time-limited clinicians.

Post-amputation, mobility deficits may be identified via patient-reported outcomes or performance-based tests that evaluate a patient’s capacity under a given set of conditions [13]. For example, post-LLA individuals demonstrate reduced functional mobility [14] and self-reported mobility and balance confidence while wearing their prosthesis [15]. Decreased walking speeds per the 10-m Walk Test and endurance per the 6-min Walk Test are found post-amputation [16]. Research in healthy adults [17] and other patient populations experiencing mobility deficits, such as older adults [18], stroke survivors [19], adults with incomplete spinal cord injury [20], and adults with knee osteoarthritis [21], suggest associations between mobility and daily physical activity in the community. While similar associations have been observed among adults with LLA [22], evidence is lacking on essential mobility constructs (such as, walking speed, aerobic endurance) that are crucial to community ambulation. Furthermore, current literature provides insufficient information regarding mobility thresholds or optimal cut-points that may help clinicians in classifying patient physical activity. Hence, the purposes of this study were (a) to examine associations between daily physical activity obtained via accelerometers and patient-reported and performance-based mobility metrics among adults with LLA, and (b) to determine whether patient-reported and performance-based mobility metrics can distinguish between physical activity status [i.e., sedentary (< 5000 steps/day) or non-sedentary (≥ 5000 steps/day)] among adults with LLA. We hypothesized mobility metrics would be associated with daily physical activity and distinguish between physical activity levels among adults post-LLA.

## Methods

### Participants

Participants for this cross-sectional study were recruited from August 2016 to September 2019 through regional clinical practices, the University of Delaware Amputee Clinic, local community healthcare practices, and databases of individuals interested in future research participation. Individuals were included if they were aged 18–85 years, English-speaking and -reading, were ≥ 1-year post-transtibial amputation (TTA) or -transfemoral amputation (TFA) and used a prosthesis to walk inside and outside their home. Participation was limited to individuals reporting a Saltin-Grimby Physical Activity Scale rating of 1 (i.e., physically inactive: doing sedentary activities during leisure time, such as, reading, watching television, using computers, etc.) or 2 (i.e., light physical activity: physical activity for at least 4 h/week, such as, riding a bicycle or walking to work, gardening, etc.) [23], with the intent of capturing individuals not meeting physical activity guidelines (less than moderate-intensity physical activity) for adults with function-limiting conditions [12]. Potential participants were excluded if they had (a) bilateral LLA, (b) experienced a recent hospitalization, (c) a systemic neuromuscular disease, or (d) a current illness or condition (e.g., limb ulcer, infection, uncontrolled blood pressure) affecting their ability to safely participate.

### Procedures

Participants provided their demographic (e.g., age, sex, height, weight) and amputation-specific (e.g., time since amputation, amputation etiology) information and completed the Houghton Scale, which evaluates prosthesis use and stability; test–retest reliability has been previously reported [intraclass correlation coefficient (ICC)_2,1_ = 0.96] [24].

### Mobility assessment

#### Patient-reported

The Prosthesis Evaluation Questionnaire-Mobility Subscale (PEQ-MS) is a reliable [ICC_3,1_ = 0.92], 12-item measure of perceived ambulation ability while wearing a prosthesis [25]. Items are scored on a 0 to 4 ordinal scale, where 4 indicates ‘no problem in completing the activity’, and summed; higher scores indicate greater prosthesis-enabled mobility [25].

#### Performance-based

The L-Test is a reliable (ICC_2,2_ = 0.96) measure that has been used to evaluate functional mobility post-LLA [26]. Participants were instructed to standup from a chair with armrests, walk three meters, turn 90°, walk seven meters, turn 180°, retrace the L-shaped path to the chair, and sit down. Following demonstration by an examiner, participants completed one timed trial (recorded in seconds).

The 10-m Walk Test (10mWT) is a reliable (ICC_1,1_ = 0.98) measure that evaluates walking speed [27]. On a straight 10-m path, participants were instructed to complete three trials at their “self-selected walking speed” (SSWS). For all trials, speed was calculated over the middle six meters, allowing for acceleration and deceleration at either end of the path. Average SSWS was used for analyses.

The 6-min Walk Test (6MWT) is a reliable (ICC_3,1_ = 0.94) measure used to assess endurance (i.e., aerobic capacity) post-LLA [28]. Participants were instructed to walk along a pre-determined path for six minutes while covering as much distance as possible. A member of the research team trailed the participant to avoid pacing and recorded the distance covered in meters using a rolling measurement tool. Standardized encouragement was provided at each minute [28].

### Physical activity assessment

Participant physical activity was measured with a StepWatch™ 3 accelerometer (Modus Health, LLC, Washington, DC) for 7-days immediately following the onsite evaluation. Between-days, test–retest reliability (ICC_3,k_ = 0.90) for the StepWatch™ among healthy adults [29] and validity as compared to manual step counts among adults with TTA [30] has previously been reported. The accelerometer was worn around the prosthetic pylon, at approximately the ankle joint, and the monitor recorded strides for each 10-s interval [15]. To assess wear compliance and capture details on atypical activity during collection period, participants completed daily logs, documenting activity monitor donning and doffing times. Total step count during the collection period was estimated from the StepWatch™ stride counts (1 stride = 2 steps). Average daily step count was calculated by dividing total step count by number of days the accelerometer was worn.

### Statistics

Analyses were conducted using SPSS Statistics 28 (IBM Corp., Armonk, New York, USA). Participant characteristics and mobility metrics were examined using one-way analysis of variance or Mann–Whitney U tests, as appropriate. Correlations between age, time since amputation, mobility metrics and physical activity were examined using Pearson’s correlation coefficient. Separate hierarchical multiple regression was conducted for each mobility metric (independent variable), with physical activity as the dependent measure. Age and time since amputation were entered in Block I. Amputation level (0 = TTA; 1 = TFA) was entered in Block II. Mobility metric was entered in Block III and interaction terms (amputation level X mobility metric) were entered in Block IV. Alpha was set to 0.050 for all analyses.

#### Optimal CUT-points

Based on prior literature, participant physical activity was dichotomized as sedentary (< 5000 steps/day) or non-sedentary (≥ 5000 steps/day) [12]. To determine each mobility metric’s ability to identify physical activity status, receiver operating characteristic (ROC) curves were created, with physical activity classification (sedentary = 1; non-sedentary = 0) as the dependent measure. ROC curve analysis assesses the diagnostic ability of a test to appropriately discriminate between two classifications, for example, sedentary or non-sedentary, as well as determine cut-points that may be used to classify patients [31]. For significant models (i.e., mobility metrics that were able to distinguish between sedentary and non-sedentary physical activity status), optimal cut-points were determined using Youden’s index [32]. Positive and negative likelihood ratios, pre-test probability, and positive and negative post-test probabilities were calculated [33, 34].

### Likelihood ratios (LRs)

1. *Positive LR* The likelihood of a positive test (e.g., a test score at or below determined cut-point for PEQ-MS, 10mWT, 6MWT, and at or above determined cut-point for L-Test) among participants with the condition of interest (i.e., sedentary) as compared to participants who do not have the condition of interest (i.e., non-sedentary). Essentially, this is the increase in odds of sedentarism with a positive test. Higher values of positive LR are desirable.
  $$Positive\;LR = \frac{Sensitivity}{{\left( {100 - Specificity} \right)}}$$
2. *Negative LR* The likelihood of a negative test (e.g., a test score at or above determined cut-point for PEQ-MS, 10mWT, 6MWT and at or below determined cut-point for L-Test) among participants with the condition of interest (i.e., sedentary) as compared to participants who do not have the condition of interest (i.e., non-sedentary). Essentially, this is the decrease in odds of sedentarism, given a negative test. Smaller values of negative LR are desirable.
  $$Negative\;LR = \frac{{\left( {100 - Sensitivity} \right)}}{Specificity}$$

### Probabilities

1. *Pre-test probability* The probability of a participant to be sedentary prior to administering any mobility test.
  $$Pretest\;probability = \frac{{N_{sedentary} }}{{N_{total} }}$$
2. Post*-test positive probability* The probability of a participant to be sedentary given a test score at or below determined cut-point for PEQ-MS, 10mWT, 6MWT, and at or above determined cut-point for L-Test. Higher values of post-test positive probability are desirable.
  $$Posttest\;positive\;probability = \frac{Pretest\;probability*Positive\;LR}{{\left[ {1 + \left( {Pretest\;probability*Positive\;LR} \right)} \right]}}$$
3. *Post-test negative probability* The probability of a participant to be sedentary given a test score at or above determined cut-point for PEQ-MS, 10mWT, 6MWT and at or below determined cut-point for L-Test. Smaller values of post-test negative probability are desirable.
  $$Posttest\;negative\;probability = \frac{Pretest\;probability*Negative\;LR}{{\left[ {1 + \left( {Pretest\;probability*Negative\;LR} \right)} \right]}}$$

Based on cut-points, participant data for each mobility metric was dichotomized as ‘risk of sedentarism’ (scored as ‘1’) or ‘no risk of sedentarism’ (scored as ‘0’). Each participant’s score on all four-mobility measures were summed to create a Composite Mobility Score (CMS). The CMS ranged from 0 to 4, with 0 indicating no risk of sedentarism, and 4 indicating risk of sedentarism according to all four-mobility measures. To determine CMS’s ability to estimate physical activity status of post-LLA, a logistic regression analysis was conducted, with physical activity (sedentary = 1; non-sedentary = 0) as the dependent measure and CMS (range: 0–4) as the independent measure.

## Results

### Participants

**Fig. 1:**
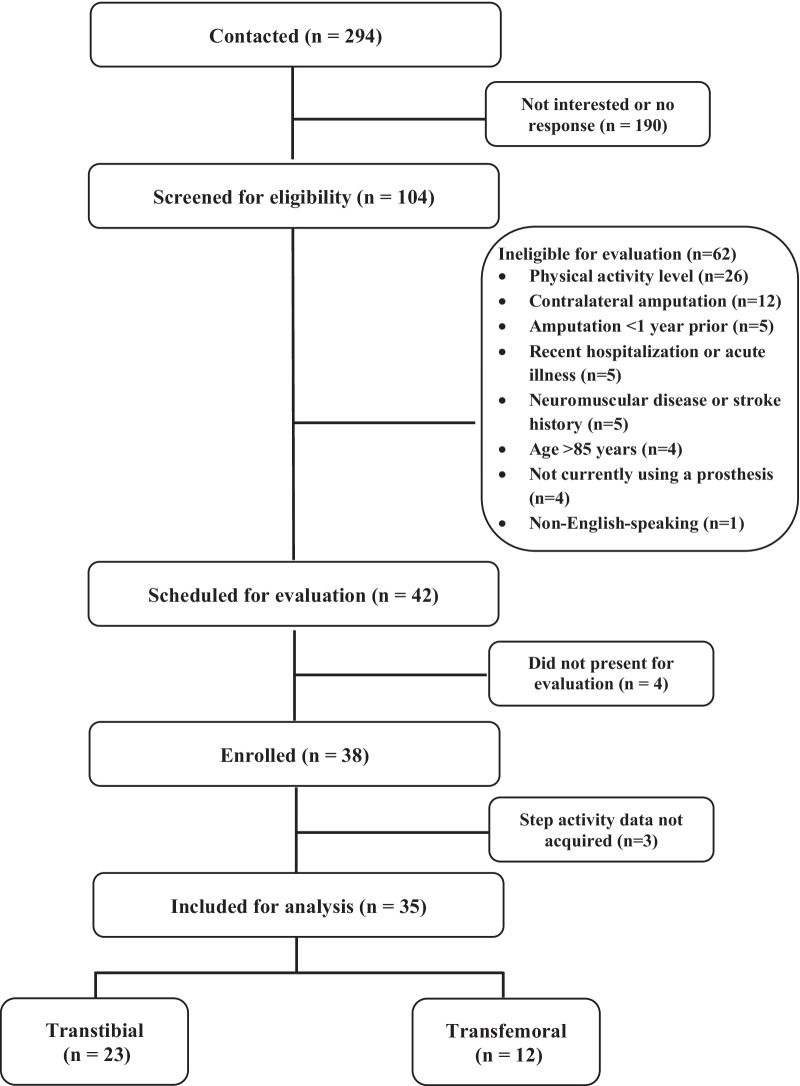
Participant selection based on the exclusion and inclusion criteria

Overall, 294 individuals were contacted for study participation. Of these, 104 were screened for eligibility (Fig. 1). Sixty-two were ineligible based on selection criteria, and 42 adults were scheduled for participation in the study. Of these, 4 individuals did not present for the scheduled evaluation and were unable to be contacted for rescheduling purposes. Thirty-eight adults with LLA (TTA, n = 24; TFA, n = 14) were enrolled in the study, however, for 3 participants, step activity data was not acquired, and participants were excluded from analysis. Hence, the study analysis included 35 adults with LLA (TTA, n = 23; TFA, n = 12).

Overall, step-activity was averaged over seven days for 27 of the 35 participants. For a subset of the sample (n = 8), step-activity was averaged over six (n = 6) or five (n = 2) days due to missing data or unexplained outliers [> ± 1.5 inter-quartile range].

Table 1 presents participant characteristics by amputation level. Participants with TTA reported significantly shorter time since amputation, and more diverse etiology distribution (Table 1). Table 2 presents participant mobility metrics by amputation level. Participants with TTA and those with TFA demonstrated similar mobility metrics (Table 2).

**Table 1:** Participant characteristics by amputation level

	Transtibial amputation (n = 23)	Transfemoral amputation (n = 12)	*p* Value
Sex (female)^†^	8 (34.8%)	5 (41.7%)	0.726
Age (years)^§^	63.0 ± 10.4	58.8 ± 9.5	0.243
Height (m)^§^	1.7 ± 0.1	1.7 ± 0.1	0.217
Weight (kg)^§^	96.0 ± 26.1	85.8 ± 18.1	0.235
Time since amputation (years)^‡^	4.0 (2.0, 10.0)	10.5 (7.5, 31.0)	0.031
Houghton Scale (0–12)^‡^	10.0 (9.0, 12.0)	10.0 (8.0, 10.8)	0.327
Physical activity (steps/day)^§^	4112 ± 2075	4244 ± 2049	0.859
Etiology^†^			
Dysvascular	7 (30.4%)	0 (0.0%)	0.026
Trauma	4 (17.4%)	6 (50.0%)	
Cancer	1 (4.3%)	2 (16.7%)	
Congenital	0 (0.0%)	0 (0.0%)	
Infection	10 (43.5%)	1 (8.3%)	
Other	1 (4.3%)	3 (25.0%)	

^†^Data presented as N (% of sample)

^§^Data presented as mean ± standard deviation

^‡^Data presented as median (25th, 75th percentile)

**Table 2:** Participant self-reported and performance-based mobility by amputation level

	Transtibial amputation (n = 23)	Transfemoral amputation (n = 12)	*Sig*
*Self-reported*			
PEQ-MS	30.7 ± 11.3	36.1 ± 11.5	0.188
*Performance-based*			
L-Test (s)	28.2 ± 9.6	31.8 ± 13.1	0.359
10mWT SSWS (m/s)	0.95 ± 0.23	0.82 ± 0.30	0.170
6MWT (m)	344 ± 115	323 ± 134	0.641

Data presented as mean ± standard deviation

*PEQ-MS* Prosthesis Evaluation Questionnaire-Mobility Subscale, *10mWT* 10-m Walk Test, *SSWS*, self-selected walking speed, *6MWT* 6-min Walk Test, *sec* seconds, *m* meters

### Regression

Participant physical activity was significantly correlated with all mobility metrics (Table 3). Interactions between amputation level and mobility metrics were not significant for any model (*p* < 0.05), therefore, only results for Block I–III are presented (Table 4). PEQ-MS score, L-Test time, 10mWT SSWS, and 6MWT distance explained 11.3%, 31.8%, 37.6%, and 30.7% of the total variance in daily step count, respectively, exceeding the variance explained by age, time since amputation, and amputation level.

**Table 3:** Correlation between mobility metrics and physical activity

	Age	TSAmp	PEQ-MS	L-Test	10mWT	6MWT	PA
Age	1						
TSAmp	0.044 (0.800)	1					
PEQ-MS	0.169 (0.333)	0.318 (0.063)	1				
L-Test	0.167 (0.338)	− 0.304 (0.076)	− 0.278 (0.106)	1			
10mWT	− 0.218 (0.208)	0.262 (0.128)	0.322 (0.059)	− 0.905* (< 0.001)	1		
6MWT	− 0.335* (0.049)	0.111 (0.524)	0.401* (0.017)	− 0.773* (< 0.001)	0.818* (< 0.001)	1	
PA	− 0.169 (0.332)	0.344* (0.043)	0.371* (0.028)	− 0.664* (< 0.001)	0.692* (< 0.001)	0.623* (< 0.001)	1

Data presented as Pearson’s r

*TSAmp* time since amputation, *PA* physical activity, *PEQ-MS* Prosthesis Evaluation Questionnaire-Mobility Subscale, *10mWT* 10-m Walk Test, *6MWT* 6-min Walk Test

*p < 0.050

**Table 4:** Regression models for physical activity

	Model 1 (PEQ-MS)		Model 2 (L-test)	
	B	*Sig*	B	*Sig*
Intercept	5196.9	0.019	7887.7	≤ 0.001
Age	− 55.0	0.102	− 10.1	0.720
TSAmp	32.0	0.115	16.3	0.356
Level	− 674.7	0.347	404.7	0.096
Mobility metric	65.5	0.039	− 117.9	≤ 0.001
R^2^	27.1%*		47.6%*	
R^2^ Change				
Block I	15.2%		15.2%	
Block II	0.6%		0.6%	
Block III	11.3%*		31.8%*	

	Model 3 (10mWT SSWS)		Model 4 (6MWT)	
	B	*Sig*	B	*Sig*
Intercept	− 1472.9	0.518	− 152.0	0.948
Age	3.1	0.910	5.8	0.847
TSAmp	14.8	0.370	31.9	0.062
Level	753.7	0.213	151.6	0.802
Mobility metric	5499.0	≤ 0.001	10.2	≤ 0.001
R^2^	53.4%*		46.5%*	
R^2^ Change				
Block I	15.2%		15.2%	
Block II	0.6%		0.6%	
Block III	37.6%*		30.7%*	

R^2^ refers to the total variance explained by the model

R^2^ change shows the variance explained by each block

Block I: Age and TSAmp; Block II: Block I + Amputation level (0 = Transtibial, 1 = Transfemoral); Block III (Model 1): Block II + PEQ-MS scores; Block III (Model 2): Block II + L-Test time; Block III (Model 3): Block II + 10mWT SSWS; Block III (Model 4): Block II + 6MWT distance

*p < 0.050 for final block

*PEQ-MS* Prosthesis Evaluation Questionnaire-Mobility Subscale, *10mWT SSWS* 10-m Walk Test self-selected walking speed, *6MWT* 6-min Walk Test, *B* unstandardized beta coefficient, *TSAmp* time since amputation, *Level* amputation level

### ROC analysis

**Fig. 2:**
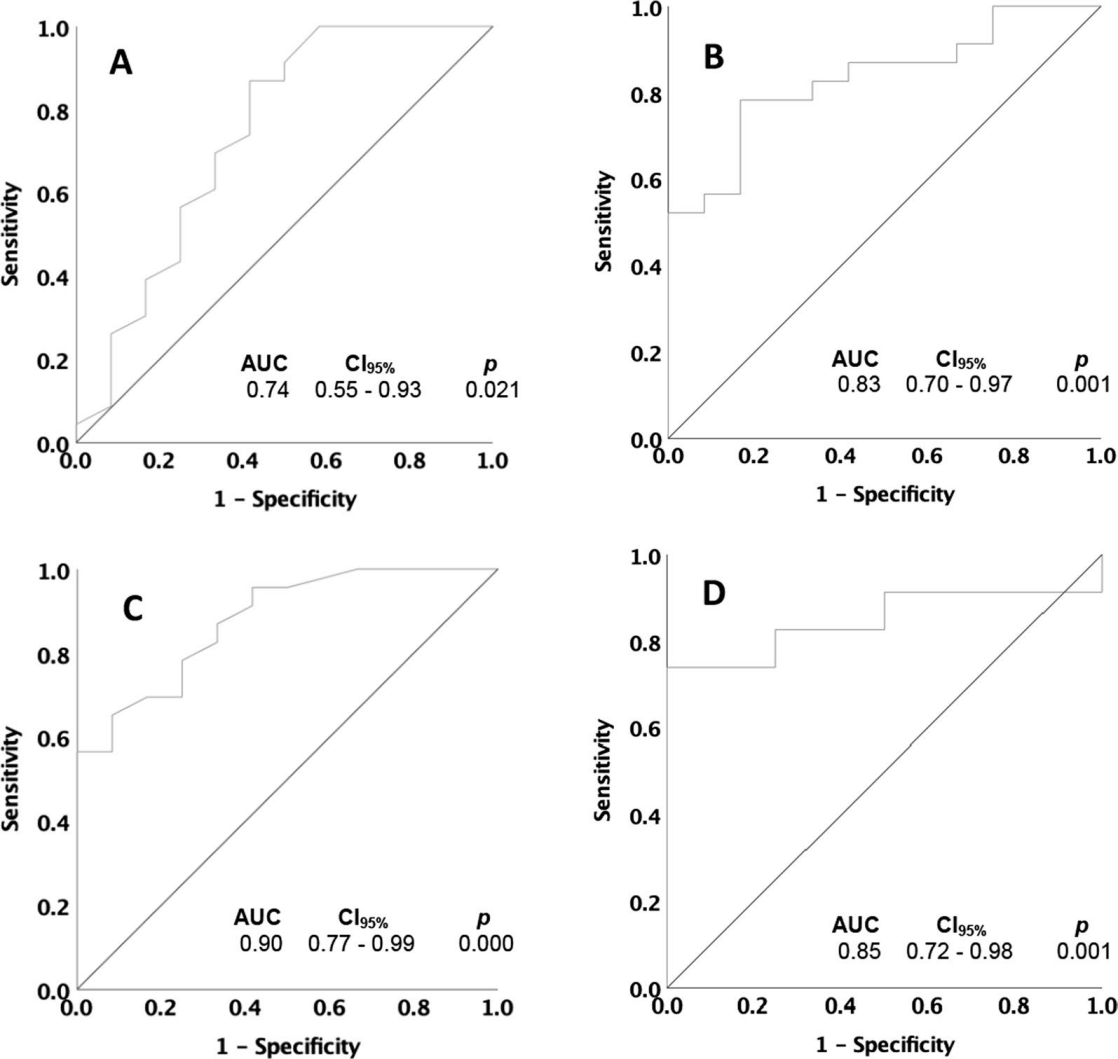
Receiver operating characteristic (ROC) curves used to calculate area under curve (AUC) and optimal cut-points for **A** Prosthetic Evaluation Questionnaire-Mobility Subscale, **B** L-Test, **C** 10-m Walk Test, and **D** 6-min Walk Test

Twenty-three of the 35 participants were classified as sedentary (i.e., walking < 5000 steps/day). The ROC analysis indicated PEQ-MS, L-Test, 10mWT, and 6MWT significantly distinguished (*p* < 0.050) between sedentary and non-sedentary adults post-LLA (Fig. 2).

#### Optimal clinical cut-points

Table 5 presents optimal clinical cut-points for PEQ-MS, L-Test, 10mWT, and 6MWT. Across all measures, the highest sensitivity (ability of the test when negative to rule-out sedentarism), i.e., 87% and lowest negative LR (decrease in odds of sedentarism, given a negative test.), i.e., 0.2, were observed for the PEQ-MS. The highest specificity (ability of the test when positive to rule-in sedentarism), i.e., 91.7%, and highest positive LR (increase in odds of sedentarism with a positive test.), i.e., 8.9, were observed for the 6MWT. The pre-test probability of the sample was 66%, and the largest increase in post-test probability for a positive test (i.e., test score at or below determined cut-point for PEQ-MS, 10mWT, 6MWT, and at or above determined cut-point for L-Test) was observed for the 6MWT (post-test positive probability of 85.4%). The largest decrease in post-test probability for a negative test (i.e., test score at or above determined cut-point for PEQ-MS, 10mWT, 6MWT and at or below determined cut-point for L-Test) was observed for PEQ-MS (post-test negative probability of 11.6%).

**Table 5:** Optimal cut-points to identify adults with LLA at risk of sedentarism

	Cut-point	Sensitivity (%)	Specificity (%)	Positive LR^†^	Negative LR^‡^	Post-test probability for positive test^§^ (%)	Post-test probability for negative test^¶^ (%)
PEQ-MS	41.0	87.0	58.3	2.1	0.2	58.0	11.6
L-test (s)	25.2	78.0	83.3	4.7	0.3	83.8	21.0
10mWT (m/s)	0.94	65.2	91.7	7.9	0.4	75.5	16.5
6MWT (m)	363.4	73.9	91.7	8.9	0.3	85.4	16.5

*LR* likelihood ratio, *PEQ-MS* Prosthesis Evaluation Questionnaire-Mobility Subscale, *10mWT* 10-m Walk Test, *6MWT* 6-min Walk Test, *m* meters, *sec* seconds

Pre-test probability of sample was 66%

^†^Ranges from 0 to infinity. Larger values indicate usefulness of measure in classifying adults at risk of sedentarism post-LLA

^‡^Ranges from 0 to 1. Smaller values indicate usefulness of measure in classifying adults not at risk of sedentarism post-LLA

^§^Probability an adult post-LLA is at risk of being sedentary given a test score at or below determined cut-point for PEQ-MS, 10mWT, 6MWT, and at or above determined cut-point for L-Test

^¶^Probability an adult post-LLA is not at risk of being sedentary given a test score at or above determined cut-point for PEQ-MS, 10mWT, 6MWT and at or below determined cut-point for L-Test

The CMS was significantly associated with participant physical activity status (*p* = 0.004), such that a 1-point increase in CMS (range: 0–4) was associated with a 6-times increased odds of a sedentary physical activity classification.

## Discussion

A loss in physical activity is anticipated following LLA, but, in the clinical environment, limited resources are available to estimate a patient’s physical activity in their home and community. Our findings indicate patient-reported and performance-based clinical mobility metrics are associated with physical activity post-LLA, such that an increase in mobility is associated with an increase in daily step activity. Moreover, following LLA, mobility metrics may be helpful in elucidating physical activity status, i.e., risk of sedentarism. Our findings expand the clinical utility of the PEQ-MS, L-Test, 10mWT, and 6MWT for patients with LLA, as these measures may be used to not only assess functional mobility, gait and fall risk [35], but may also be used to screen for physical activity status.

Performance-based mobility was linearly associated with daily physical activity post-LLA, after controlling for demographic and amputation-specific factors (Table 4). Hence, regardless of participant age, time since amputation or their amputation level (TFA or TTA), poor performance on the L-Test, 10mWT, and 6MWT was associated with lower average steps/day. Based on our findings, a 1 m/s (or 0.1 m/s) reduction in SSWS, as assessed with the 10mWT, was associated with 5499 fewer steps (or 550 fewer steps) walked each day; a 1-s increase in time to complete the L-Test was associated with 117.9 fewer steps walked each day; and 1-m reduction in distance walked on the 6MWT was associated with 10.2 fewer steps walked each day (Table 4). Our findings are consistent with prior evidence post-LLA (N = 46; age = 55.2 ± 5.8 years; time since amputation = 13.6 ± 11.1 years), where Parker et al. observed a similar association between mobility [per the 2-Minute Walk Test (2MWT)] and physical activity (evaluated with an accelerometer), beyond age and amputation level (ρ = 0.78, *p* < 0.001) [22]. Findings further align with previously observed significant bivariate relationships between physical activity (assessed as steps/day) and mobility [per the Timed Up and Go test (r =  − 0.442) and 2MWT (r = 0.404)] post- LLA [36]. Collectively, findings support the use of performance-based mobility metrics to estimate physical activity following LLA.

Patient-reported mobility with a prosthesis was linearly associated with daily physical activity post-LLA, after controlling for demographic and amputation-specific factors (Table 4), such that lower scores on the PEQ-MS were associated with lower average steps/day. Findings are consistent with previous literature, where self-reported mobility (assessed with the Locomotor Capabilities Index) was associated with physical activity (assessed as steps/day; ρ = 0.64, *p* < 0.001) among adults post-LLA (N = 46; age = 55.2 ± 5.8 years; time since amputation = 13.6 ± 11.1 years) [22]. Hence, post-LLA, individuals’ perception of their ability to ambulate with a prosthesis may estimate overall physical activity.

In addition to the observed relationship between mobility metrics and physical activity, the identified cut-points, albeit preliminary (Table 5), may allow clinicians to screen for risk of sedentarism post-LLA. For example, a patient walking 390 m during the 6MWT may not be at risk of sedentarism [given the cut-point of 363 m (Table 5)] and may be anticipated to walk > 5000 steps/day. To “rule-out” sedentarism post-LLA, clinicians may consider using any one of the four mobility measures, given negative likelihood ratios approaching 0 for each measure (Table 5). In contrast, for “ruling-in” sedentarism, only 10mWT-speed and 6MWT-distance may be appropriate in isolation, given positive likelihood ratios > 5 (which have a moderate to high likelihood of classifying sedentary participant as sedentary). It is, however, noteworthy that adults post-LLA already have a 66% probability of being sedentary (i.e., pre-test probability), and using a single mobility measure may only increase probability of correctly classifying sedentary patients to 85.4% (i.e., post-test positive probability; Table 5: 6MWT) and only decrease probability of incorrectly classifying non-sedentary patient as sedentary to 11.6% (i.e., post-test negative probability; Table 5: PEQMS). Hence, to gain better confidence in classifying physical activity status, clinicians may consider completing all four measures and using the CMS. With the CMS (range: 0–4), each one-point increase in score is associated with a 6 × times increase in the odds of being sedentary. For example, a patient that classifies as sedentary only on one measure (CMS = 1), is 18 times less likely to be sedentary compared to a patient that classifies as sedentary on all four measures (CMS = 4).

Physical activity screening, in addition to identifying risk of sedentarism post-LLA, may enhance prosthesis prescription. Patient prosthesis prescription, as well as component reimbursement, may be based on a clinician’s classification of the patient’s functional mobility level, where higher levels are associated with greater ambulatory potential and activity participation [37]. Objective estimation of physical activity level may mitigate clinician biases and reduce inter-rater variability when determining functional mobility classification. Given prosthesis prescription is influenced by functional mobility classifications [11], increased objectivity may be vital to mitigating healthcare disparities and promoting health equity.

Physical activity is essential to maintenance of health and lowering risk of all-cause mortality [3]. In light of our findings and prior research [22, 36], mobility and physical activity appear to be intimately connected following LLA. Incorporation of standard mobility metrics during clinical examinations may provide objective estimates of mobility status *and* physical activity. Aligning with prior research post-LLA [3, 4], the majority of study participants, i.e., 23 of 35, were sedentary, but not all were, despite their self-report [23]. Accurate, objective classification of physical activity status may support physical activity-related treatment selections and prioritization. Further, the ability to quickly assess for sedentarism with clinical measures enables clinicians to invest time required for critical communication, such as motivational interviewing, necessary to facilitate behavioral lifestyle changes [38], or behavioral counseling, which has been shown to be effective for increasing physical activity in adults with chronic health conditions [39].

### Study strengths and limitations

While preliminary cut-points for estimating physical activity status post-LLA using clinical mobility measures are provided, future, larger-scale studies may consider establishing cut-points by amputation level (i.e., TTA, TFA) or other amputation-specific (e.g., etiology, time since amputation) characteristics [40]. Moreover, longitudinal examination of mobility and physical activity post-LLA is necessary to evaluate relationships beyond associations, i.e., causation. Future studies may also consider exploring cumulative mobility scales with fewer than 4 mobility metrics, to potentially reduce the time necessary to estimate patient physical activity.

Findings may not be generalized to adults with recent LLA (i.e., < 1 year), those with bilateral amputations, minors, or adults with LLA participating in moderate-intensity activity (i.e., ≥ 2 h/week) during their leisure time. Physical activity assessed as steps/day using StepWatch accelerometers, while valid as compared to manual step counts in a controlled environment, may not accurately capture complex movement patterns required for community negotiations, nor duration or intensity of physical activity bouts. Hence, findings may not be generalizable to individuals participating in short bouts of intense physical activity. Moreover, physical activity was dichotomized as sedentary (< 5000 steps/day) and non-sedentary (≥ 5000 steps/day) based on previous guidelines for adults with chronic health conditions [12], however, post-LLA other physical activity levels (e.g., 6500 steps/day) [41] may need to be considered.

## Conclusions

Following a unilateral LLA, mobility metrics may enable clinicians to estimate physical activity. Based on our findings, greater patient-reported and performance-based mobility are associated with greater daily step activity in the home and community. Moreover, mobility metrics can distinguish adults with LLA who are sedentary (< 5000 steps/day) from peers who are non-sedentary (i.e., ≥ 5000 steps/day), and hence, mobility metrics may be clinically useful for identifying risk of sedentarism, precluding the need for step activity monitoring. Physical activity classification may support clinical decisions regarding treatment selections and prioritization, as well as prosthesis prescription. Future research may confirm findings in a larger, more diverse sample of adults with LLA, while considering amputation etiology and other potentially relevant factors. Further, longitudinal investigations may highlight mobility changes overtime that may influence physical activity post-LLA.

## Data Availability

The datasets used and analyzed during the current study are available from the corresponding author on a reasonable request and upon completing a data sharing agreement. The data are not publicly available due to inclusion of unique health information from participants in a specific and limited region that could compromise participant confidentiality.

## Abbreviations

LLA: Lower-limb amputation
TTA: Transtibial amputation
TFA: Transfemoral amputation
ICC: Intraclass correlation coefficient
PEQ-MS: Prosthesis Evaluation Questionnaire-Mobility Subscale
10mWT: 10-m Walk Test
6MWT: 6-min Walk Test
ROC: Receiver operating characteristic
CMS: Composite Mobility Score
